# Diet-Associated Gut Bacterial Microbiota and Metabolome Signatures Linked to Fermented Food Intake in Healthy Postmenopausal Women

**DOI:** 10.3390/foods15071210

**Published:** 2026-04-02

**Authors:** Natthanan Buranavanitvong, Chayaporn Thanthithum, Kanyarat Kanyakam, Dalila Azzout-Marniche, Delphine Jouan-Rimbaud Bouveresse, Nattida Chotechuang, Cheunjit Prakitchaiwattana

**Affiliations:** 1 Department of Food Technology, Faculty of Science, Chulalongkorn University, Patumwan, Bangkok 10330, Thailand; 2 UMR Physiologie de la Nutrition et du Comportement Alimentaire, Institut National de Recherche pour l’Agriculture, l’Alimentation et l’Environnement (INRAE), AgroParisTech, Université Paris-Saclay, 91120 Palaiseau, France

**Keywords:** gut bacterial microbiota, gut metabolome, vegetarians, omnivores, fermented plant-based food, SCFAs, EAAs

## Abstract

Long-term adherence to plant-based diets can modify gut bacterial microbiota composition and metabolite profiles, which may be particularly relevant for postmenopausal women who frequently adopt such diets and experience age-related changes in nutrient absorption and metabolism. Fermented foods, commonly consumed in vegetarian diets, enhance dietary diversity and nutritional quality. This study compared gut bacterial microbiota and fecal metabolomes between vegetarians (VGs) and omnivores (OMs) and evaluated the contribution of fermented food intake. Thirty-two healthy postmenopausal Thai women (>55 years; 16 VGs, 16 OMs) were enrolled. Gut bacterial microbiota and fecal metabolites were analyzed using 16S rRNA metagenomic and untargeted ^1^H-NMR metabolomics. The five most frequently consumed fermented foods were microbiologically characterized. Fermented food consumption was found to be significantly different between groups. OM participants reported infrequent consumption (<10% per week), whereas VG participants consumed fermented foods daily, often in multiple forms (>60% of weekly meals). VG participants exhibited enrichment of *Prevotella*, *Faecalibacterium*, and *Blautia*, while OM participants showed higher abundances of *Bacteroides* and *Escherichia*–*Shigella*. LEfSe identified *Weissella* as a bacterial taxon associated with the VG group. Functional prediction and metabolomic analyses indicated enhanced carbohydrate fermentation and increased short-chain fatty acid (SCFA) production in VGs, whereas OM profiles reflected greater protein catabolism. Fermented foods consumed by VGs shared microbial biomarkers with the VG gut bacterial microbiota and were rich in SCFAs and essential amino acids, supporting their potential role as microbial and metabolic contributors within the gut ecosystem and nutritional adequacy in postmenopausal vegetarians.

## 1. Introduction

The human gastrointestinal tract is inhabited by complex microbial communities collectively known as the gut microbiota. This complex ecosystem comprises more than 100 trillion microbial cells, including bacteria, archaea, fungi, helminths and protozoa [1]. The human gut microbiota, defined as the total of all microbial taxa associated with human beings (bacteria, viruses, fungi, protozoa, archaea), consists of a newly estimated 3 × 10^13^ taxa. The gut bacterial microbiota is primarily dominated by two major phyla: Bacteroidetes—including *Bacteroides* and *Prevotella*—and Firmicutes, which includes genera such as *Clostridium*, *Enterococcus*, *Lactobacillus*, and *Fecalibacterium*. Other phyla, such as Actinobacteria (mainly *Bifidobacterium*), Proteobacteria, Verrucomicrobia, and archaea contribute minorly [2,3].

Recent research data and data analysis methods have revealed functions of the human gut bacterial microbiota which are increasingly recognized as critical modulators of human health and well-being. They play essential roles in digestion, metabolism, immune regulation, and chronic conditions, including metabolic syndrome, neurodegenerative diseases, osteoporosis, hypertension, cardiovascular disease, and cancer [4,5]. Numerous studies have demonstrated that factors such as age, host genetics, antibiotic exposure, and dietary patterns can influence the balance, composition and functionality of the gut bacterial microbiota. These factors may lead to temporary or long-term alterations in microbial diversity, abundance, and metabolic pathways [2,5]. Among them, dietary patterns represent a particularly important and modifiable factor capable of significantly shaping both the gut bacterial microbiota and the produced metabolites [6,7].

Plant-based diets, including vegan and vegetarian dietary patterns, have gained global popularity in recent decades, with an estimated one billion individuals adopting such vegetarian lifestyles [8]. While vegetarians typically exclude meat, vegans avoid all animal-derived products. These diets are characterized by a lower intake of energy, saturated fat, and cholesterol, alongside a higher intake of dietary fiber, carotenoids, vitamins, potassium, magnesium, and health-promoting phytochemicals. In contrast, omnivorous diets include both animal-derived proteins and fats [4,9]. Nevertheless, long-term plant-based diets may lead to potential deficiencies in key micronutrients, such as vitamin B12, vitamin D, calcium, zinc, and long-chain omega-3 fatty acids, as well as essential amino acids (EAAs) [10].

Extended adherence to vegan diets can increase the risk of nutrient inadequacy, a concern particularly relevant for postmenopausal women due to hormonal decline, metabolic alterations, and heightened susceptibility to noncommunicable diseases [11,12,13]. Menopause is also accompanied by gut bacterial microbiota shifts that may impair nutrient absorption and reduce the production of health-related metabolites. To enhance dietary variety and sensory appeal, long-term vegetarians and/or vegans often incorporate fermented foods, which improve flavor while producing bioactive compounds and beneficial metabolites and reducing antinutritional factors. Although taste and variety are the main reasons for their inclusion, these foods are also rich in live beneficial microorganisms and metabolites, which can support nutrient absorption, restore gut microbial balance, and promote metabolic health. Fermentation may further increase nutrient content, including vitamin B bioavailability, and in some cases, contribute to the synthesis of essential amino acids [14]. Including fermented foods in plant-based diets may therefore help mitigate nutrient deficiencies and support overall health in postmenopausal vegetarian women. Notably, fermented foods contain functional microbes such as *Lactobacillus*, *Bifidobacterium*, and *Saccharomyces*, which help support and balance the gut microbiota. These microorganisms contribute to the biosynthesis of essential nutrients by fermenting complex carbohydrates and proteins into oligosaccharides and amino acids, respectively. They are also involved in the production of vitamins (such as B12 and folate), bioactive compounds with anti-inflammatory and antioxidant properties, short-chain fatty acids (SCFAs) that support gut barrier integrity, and EAAs, which are often limited in plant-based protein sources. Moreover, fermented foods can reduce antinutritional factors (ANFs) by degrading compounds such as phytic acid, tannins, and lectins, leading to improved nutrient availability and digestibility [14,15].

Diet–bacterial microbiota interactions have gained increasing attention. However, few studies have examined how fermented foods influence gut microbial composition and metabolite production in postmenopausal populations. The relationships between fermented food intake, gut bacterial microbiota, metabolite profiles, nutrient status, and health outcomes remain poorly understood. Understanding differences in gut bacterial microbiota and metabolomes between postmenopausal vegetarians and omnivores could help identify microbial and metabolic biomarkers that support nutrient biosynthesis and prevent deficiencies. In Thailand, several vegetarian communities have maintained consistent dietary patterns for over 10 years, preparing traditional fermented foods from rice, soy, mushrooms, and vegetables such as sweetened fermented rice (Kaow-mark), salty fermented soybean paste (Kapi-Jae), fermented soybean milk (soy yogurt), sour fermented mushrooms (Nham Hed), and sour fermented vegetables. These products are typically consumed fresh, without thermal processing, and are integrated into daily meals to enhance flavor and palatability.

The present study aims to assess the potential of fermented foods to promote health via modulation of the gut bacterial microbiota and metabolome in healthy postmenopausal women from vegetarian communes, in comparison with postmenopausal omnivorous women. The gut bacterial microbiota and metabolome profiles of participants and the fermented foods they consume were analyzed using metagenomic and metabolomic analysis and bioinformatic approaches. The findings could reveal how microorganisms and metabolites from fermented foods interact with the gut bacterial microbiota of consumers, helping to determine whether current dietary practices confer health benefits or could be optimized. These findings may also identify novel microbial or metabolic signatures that can be applied to develop targeted foods, functional ingredients, or probiotic and prebiotic supplements derived from these fermented foods.

## 2. Materials and Methods

### 2.1. Participants and Study Design

This study was conducted using an observational cohort design. Participants were screened using a questionnaire from an initial pool of more than 60 individuals. The inclusion criteria specified that VGs has followed a vegetarian diet for at least 5 years and strictly avoided all meat, poultry and seafood. The inclusion criteria for all participants included an age > 55 years and a body mass index (BMI) < 25 kg/m^2^ and a weekly consumption of fermented foods. The exclusion criteria were related to metabolism, diseases of the digestive tract, antibiotic therapy in the past 3 months, and any chronic medication (including medications for diabetes, hypertension and cardiovascular disease), and regular (daily) smoking and alcohol consumption. Each participant completed questionnaires to assess participant information including age, body weight, height, health disease status, fermented food frequency and dietary patterns, and feces were collected [10,16,17]. The sample size in this study was designed to investigate the associations of a long-term vegetarian diet compared to an omnivorous diet. The sample size provided 80% power to detect a difference with an effect size 0.6 at alpha 0.05 using two-sided paired *t*-tests. The number of participants in each group was calculated by G*Power version 3.1.9.6 [16,18].

To minimize potential bias, the study was conducted in a single-blind manner. In addition, eligible participants were assigned identification numbers, and all analyses were performed using anonymized datasets.

#### Study Approval

All the participants gave informed consent prior to enrollment. The research protocol was approved by The Research Ethics Review Committee for Research Involving Human Research Participants, Group I, Chulalongkorn University (CoA No. 072/67 and 060/68) in accordance with the Declaration of Helsinki. The study was registered with the Thai Clinical Trial Registry under the number TCTR20240619003 (https://www.thaiclinicaltrials.org/show/TCTR20240619003, accessed on 10 May 2024).

### 2.2. Sample Collection

#### 2.2.1. Fecal Sample Collection

Fecal samples were collected during a habitual dietary period for the participants in both the VG and OM groups. Individuals in the VG group had followed a vegetarian dietary pattern at least 5 years and reported habitual consumption of plant-based fermented foods as part of their usual diet prior to enrollment. Fecal samples (5 g) were collected from both the VG and OM groups using sterile containers. For metagenomic analysis, 0.5 g of each sample was transferred into DNA/RNA Shield™ Fecal Collection Tubes (Zymo Research Corp., Irvine, CA, USA) and thoroughly homogenized by vortex mixing. The samples were then transported and processed for 16S rRNA-based metagenomic analysis at Mod Gut Co., Ltd. (Bangkok, Thailand) [17]. For metabolomic analysis, 1.0 g of each fecal sample was transferred into sterile Eppendorf tubes. All samples were immediately placed on ice during transportation and stored at −80 °C until further analysis.

#### 2.2.2. Fermented Food Collection

The bacterial microbiota and metabolite profiles were investigated by collecting several fermented food samples from the vegetarian (FVG) and omnivore group (FOM) through interviews. The five most frequently consumed fermented foods in the VG group included sweetened fermented rice (Khao-Mak; FVG-1), a salty fermented soybean condiment (plara-jae; FVG-2), sour fermented mushrooms (nham: FVG-3), salty fermented soybean paste (kapi-jea: FVG-6), and sour fermented spinach (FVG-7). Four FOMs were fermented soybean curd (FOM-1), sour fermented spider flower (pak sian dong; FOM-2), salty fermented fish (FOM-3), and kefir (FOM-4). The samples were immediately placed on ice during transportation to the laboratory. Of each sample, 1.0 g was subjected to cultural plating to count the viable microbial cells [19]. Five grams of each sample was transferred to sterile tubes for metagenomic analysis, and 1.0 g of each sample was transferred to sterile Eppendorf tubes for metabolomic analysis. All samples were stored at −80 °C until further analysis.

### 2.3. Metagenomic Analysis

#### 2.3.1. DNA Extraction and Sequencing

Microbial profiling was performed using 16S rRNA gene sequencing, which targets bacterial taxa. The samples were extracted for DNA by the ZymoBIOMICS™ DNA Miniprep Kit (Zymo Research Corp, Irvine, CA, USA). The quality and concentration of the extracted DNA were assessed using a DeNovix™ UV-Vis spectrophotometer (DeNovix Inc., Wilmington, DE, USA). Then, amplicon-based 16S rRNA gene sequencing was amplified using the forward primer 515 F (5′- GTG CCA GCM GCC GCG GTA A -3′) and the reverse primer 806R (5′- GGA CTA CHV GGG TWT CTA AT -3′). Paired-end sequencing was performed on the Illumina MiSeq 2 × 300 bp platform (San Diego, CA, USA) [17,20].

#### 2.3.2. Bacterial Microbiota Processing and Analysis

Bacterial microbiota bioinformatics was performed using QIIME 2 (version 2024.2). The raw sequencing data were demultiplexed using the q2-demux plugin and denoised using DADA2. The amplicon sequence variants (ASVs) were identified using the SILVA reference database with a sequence similarity threshold of >99%. A rarefied table with a sampling depth of 30,000 sequences was used to calculate alpha and beta diversity metrics. The data was uploaded to the MicrobiomeAnalyst online platform (www.microbiomeanalyst.ca, accessed on 2 July 2025) for Marker Data Profiling and advanced visualization [21,22].

#### 2.3.3. Functional Annotation

The metabolic functions and pathways of the microbial communities in each group were predicted using PICRUSt2 software version 2.5.1 (Phylogenetic Investigation of Communities by Reconstruction of Unobserved States) based on the reference genome annotations in KEGG (Kyoto Encyclopedia of Genes and Genomes) pathways. STAMP software version 2.0 (Statistical Analysis of Metagenomic Profiles) was used to compare differences in the predicted metabolic function abundances between groups [20,23].

### 2.4. Metabolomic Analysis

Sample preparation and metabolome analysis by nuclear magnetic resonance (NMR) spectroscopy, following a previous publication, was conducted [24,25]. A weight of 0.5 g feces and 1.0 g fermented foods from the VG and OM groups after storage at −80 °C were thawed and extracted at ratios of 1:2 (weight of fresh feces to buffer) in 0.1 M phosphate-buffered saline (PBS) at pH 7.4. The samples were mixed and vortexed for 10 min and then centrifuged at 15,000 *g* for 20 min at 4 °C. The supernatant (500 µL) was transferred into an Eppendorf tube for ultrafiltration through a 3 kDa molecular weight cutoff (Pall Nanocep^®^, Pall Life Sciences, Ann Arbor, MI, USA). The filtrate was mixed in a 1:1 ratio with buffer consisting of 300 mM KH_2_PO_4_ + 10% (*w*/*w*) deuterium oxide (D_2_O) and 1 mM 3-(Trimethylsilyl) proprionic-2, 2, 3, 3-d4 acid sodium salt (TSP) at pH 7.5 as the internal standard [26,27]. Finally, 600 µL of the mixture was transferred and subjected to analysis with a Bruker Avance III HD 500 MHz NMR spectrometer (Bruker, Rheinstetten, Germany). The ^1^H NMR spectra were aligned and calibrated based on the internal standard peak. For each spectrum, chemical shift (d) across a range of 0.00–10.00 ppm was segmented (binning) with an interval of 0.02 ppm and the signal intensity in each bin was integrated using Topspin (V 4.0.7, Bruker Biospin, Rheinstetten, Germany) to derive a quantity for each spectrum. A metabolite spectrum identification was assigned according to the ChenomxNMR suite 10.0 library (Chenomx Inc., Edmonton, AB, Canada). The identified metabolites were classified into 8 chemical classes: amines, amino acids and derivatives, organic acids and derivatives, nucleotides and derivatives, lipids and derivatives, vitamins, sugars and derivatives, and others [28,29].

### 2.5. Statistical Analysis

Bacterial microbiota analysis: Statistical tests for the alpha and beta diversity analyses were performed using QIIME 2. Alpha diversity was assessed using the Chao1, Observed, Shannon, and Abundance-based Coverage Estimator (ACE) indexes, with statistical significance determined by the Kruskal–Wallis test. Beta diversity was evaluated using the Bray–Curtis and Jaccard indices and compared between groups using Permutational Multivariate Analysis of Variance (PERMANOVA). The microbial composition was further explored through interactive stacked bar plots and heatmaps to visualize taxonomic abundance and correlation patterns. A Linear Discriminant Analysis Effect Size (LEfSe) analysis was performed to identify significantly differentially abundant microbial biomarkers between groups. Initially, nonparametric Kruskal−Wallis tests were applied to detect significant features, followed by Linear Discriminant Analysis (LDA) to estimate the effect size of each differentially abundant feature. The results were supported by 30-fold bootstrapping, with a logarithmic LDA score cutoff of ≥1.0 to determine biological relevance. Statistical analysis of the functional annotations was performed using Welch’s *t*-test with a 95% confidence interval.

Metabolomic analysis: The metabolite analyses were performed using the MetaboAnalyst 6.0 online platform (www.metaboanalyst.ca, accessed on 17 September 2025). The metabolomic data were normalized by sample median, followed by autoscaling (mean centering and division by the standard deviation of each variable). Ward’s linkage method was used for hierarchical clustering, and appropriate statistical tests were applied within the platform.

The participants’ characteristics were presented as mean ± standard deviation (SD) using SPSS (version 22.0.0, SPSS Inc., Chicago, IL, USA). The independent samples *t*-test and one-way ANOVA were performed to compare the means between groups, as appropriate. Statistically significant differences were considered at a significance level of *p* < 0.05.

## 3. Results

**Figure 1:**
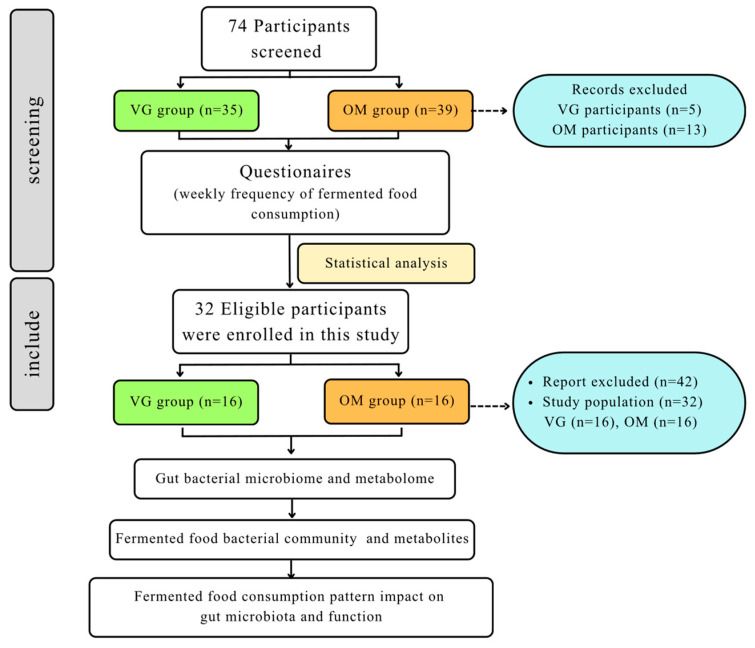
Flow diagram of participant selection and study design.

The screening of the 74 participants was conducted using predefined inclusion and exclusion criteria through structured questionnaires. Of these, 60.68% met the health-related inclusion and exclusion criteria. The eligible participants subsequently completed an additional questionnaire assessing the weekly frequency of fermented food consumption. The results indicated that the frequency of fermented food intake in the vegetarian group (VG) was significantly higher than that in the omnivorous group (OM). Based on the statistical sample size calculation, the minimum acceptable number of participants was determined to be 16 per group. Therefore, participants in each group were selected by ranking the frequency of fermented food consumption, and the first 16 participants with the highest frequency in each group were included for analysis (Figure 1).

**Figure 2:**
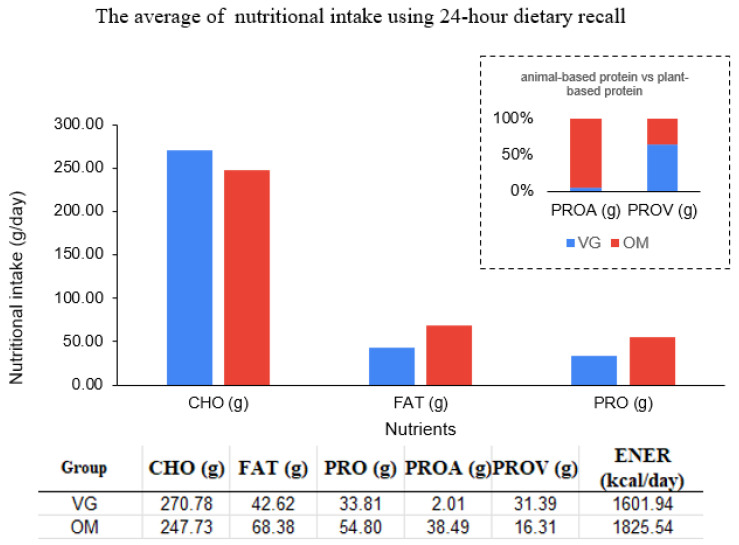
The average habitual nutritional intake pattern of the vegetarian (VG) and omnivore (OM) groups. (CHO; carbohydrate, PRO; protein, PROA; animal-based protein, PROV; plant-based protein).

The cross-sectional study included 32 postmenopausal participants with 16 vegetarians (VGs) and 16 omnivores (OMs). Eligible participants in the VG group had a mean age of 63.87 ± 4.33 years and a mean BMI of 19.79 ± 2.03 kg/m^2^, whereas those in the OM group had a mean age of 62.06 ± 5.74 years and a mean BMI of 21.48 ± 1.61 kg/m^2^. There were no significant differences in age or BMI between the groups (*p* > 0.05). The estimated habitual nutritional intake analysis through INMUCAL revealed the VG group consumed more carbohydrates (VG:OM 270.78:247.73 g per day), while the OM group had a significantly higher intake of fat and protein (OM:VG 68.38:42.62, and 54.80:33.81 g per day, respectively) (Figure 2). Fecal samples from both groups were collected for analysis. In addition, fermented food products were collected, and data on daily intake and consumption patterns were documented.

### 3.1. Pivotal Interplays of Key Diversity and Functional Pathways Between VG and OM Gut Bacterial Microbiota

The gut bacterial microbiota diversity within individuals (alpha diversity) was assessed to evaluate the richness and evenness of microbial communities in the vegetarian (VG) and omnivore (OM) groups. Four indices were used: Chao1, which estimates species richness by predicting the number of Operational Taxonomic Units (OTUs); ACE, which estimates overall richness, including rare taxa; Observed, representing the actual number of unique OTUs; and Shannon, reflecting both species richness and evenness based on the relative abundance of taxa. Fecal samples from both groups, along with fermented food products, were collected for analysis, and data on daily intake and consumption patterns were documented. Statistical comparisons of alpha diversity were performed using the Mann–Whitney/Kruskal–Wallis test [20].

**Figure 3:**
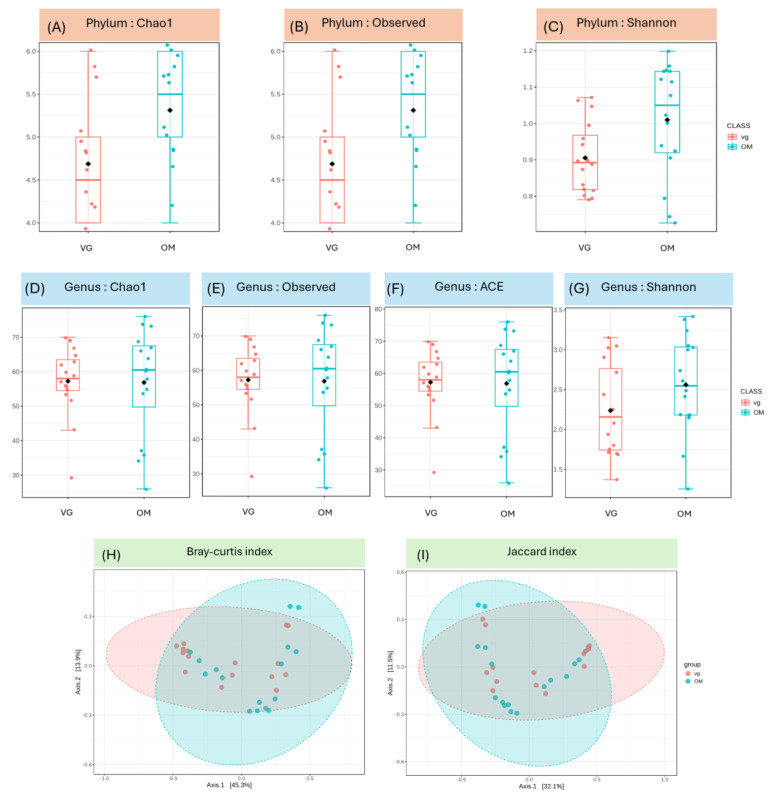
Alpha and beta diversity analysis of gut bacterial microbiota in vegetarian (VG) and omnivorous (OM) groups. Alpha diversity was evaluated at the phylum level using the (**A**) Chao1, (**B**) Observed, and (**C**) Shannon indices and at the genus level using the (**D**) Chao1, (**E**) Observed, (**F**) Shannon, and (**G**) ACE indices. Statistical comparisons between groups were performed using the Kruskal–Wallis test. Beta diversity analysis at the genus level was performed using the (**H**) Bray–Curtis and (**I**) Jaccard indices. Differences in community composition between groups were tested using PERMANOVA (*p* < 0.05).

At the phylum level, the OM group exhibited significantly higher microbial diversity than the VG group (Chao1: *p* = 0.0377; Shannon: *p* = 0.0377; Observed: *p* = 0.0288; Figure 3A–C), whereas no significant differences were observed at the genus level (Chao1, Observed, ACE, Shannon; *p* = 0.6646, 0.6646, 0.6646, 0.1486; Figure 3D–G). Although phylum-level diversity differed, genus-level alpha diversity remained stable.

The beta diversity of the microbial communities illustrates the relative differences in species composition between VGs and OMs. Beta diversity analysis revealed statistical separation between the VG and OM groups in Principal Coordinate Analysis (PCoA) plots based on Bray–Curtis and Jaccard distances. PERMANOVA confirmed that these differences were significant for both distance metrics (*p* = 0.044 and 0.046, respectively; Figure 3H,I), indicating that dietary patterns exert a notable influence on overall gut microbial community structure.

**Figure 4:**
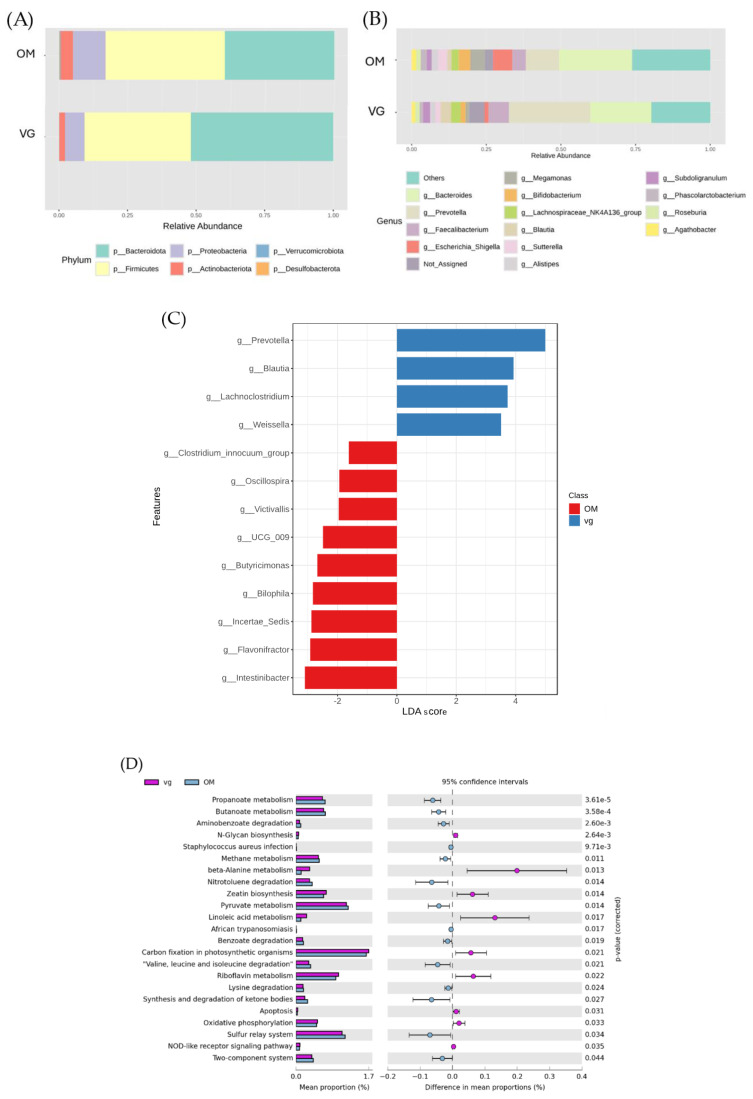
Bacterial taxonomic composition and functional prediction of gut microbiota in vegetarian (VG) and omnivorous (OM) groups. (**A**) Relative abundance of bacterial taxa at the phylum level. (**B**) Relative abundance of dominant bacterial taxa at the genus level. (**C**) Differentially abundant bacterial genera identified using Linear Discriminant Analysis Effect Size (LEfSe) analysis, with LDA scores. (**D**) Predicted functional pathways of the gut bacterial microbiota based on KEGG pathway analysis.

Bacterial taxonomic profiling supported these findings, revealing distinct compositional differences between the two dietary groups. At the phylum level, Bacteroidota and Firmicutes predominated in both groups. Bacteroidota were more abundant in VGs (51.93%) than in OMs (39.78%), whereas Firmicutes were slightly higher in OMs (43.34% vs. 38.73%). Other phyla, including *Proteobacteria*, *Actinobacteria*, *Verrucomicrobiota*, and *Desulfobacterota*, were present at lower proportions (Figure 4A).

At the genus level, dietary influences were pronounced. The VG group was dominated by *Prevotella* (28.2%), *Bacteroides* (20.1%), *Faecalibacterium* (6.7%), *Blautia* (3.4%), and *Lachnospiraceae*_NK4A136 (3.09%). The OM group was characterized by *Bacteroides* (25.4%), *Prevotella* (11.2%), *Escherichia–Shigella* (6.5%), *Megamonas* (4.9%), *Bifidobacterium* (4.6%), and *Sutterella* (3.1%), as shown in Figure 4B.

Linear Discriminant Analysis Effect Size (LEfSe) was performed to identify genera that distinctly characterize each dietary group. As shown in Figure 4C and Figure S1, the VG group was significantly enriched in *Prevotella*, *Blautia*, *Lachnoclostridium*, and *Weissella* (*p* < 0.05), consistent with plant-based, high-fiber diets. In contrast, the OM group exhibited higher abundances of *Intestinibacter, Flavonifractor, Incertae Sedis, Bilophila*, and *Butyricicoccus* (*p* < 0.05), genera often linked to higher intake of animal-based foods and fats.

### 3.2. Functional Pathway Annotations of Gut Bacterial Microbiota and Fecal Metabolites

The predicted functional pathway annotations, inferred from PICRUSt-based KEGG pathway enrichment analysis, identified 23 metabolic pathways, highlighting clear distinctions in microbial functional potential between the VG (vegetarian) and OM (omnivore) groups (*p* < 0.05), as shown in Figure 4D, reflecting the impact of long-term dietary patterns on the predicted metabolic functions of the gut bacterial microbiota metabolism. In the VG group, pathways such as beta-alanine metabolism, linoleic acid metabolism, riboflavin metabolism, zeatin biosynthesis, and carbon fixation in photosynthetic organisms were enriched.

In contrast, the OM group showed enrichment in pathways such as the sulfur relay system, ketone body metabolism, degradation of aromatic compounds (e.g., nitrotoluene), branched-chain amino acid degradation (valine, leucine, isoleucine), pyruvate metabolism, and short-chain fatty acid metabolism (propanoate and butanoate).

**Figure 5:**
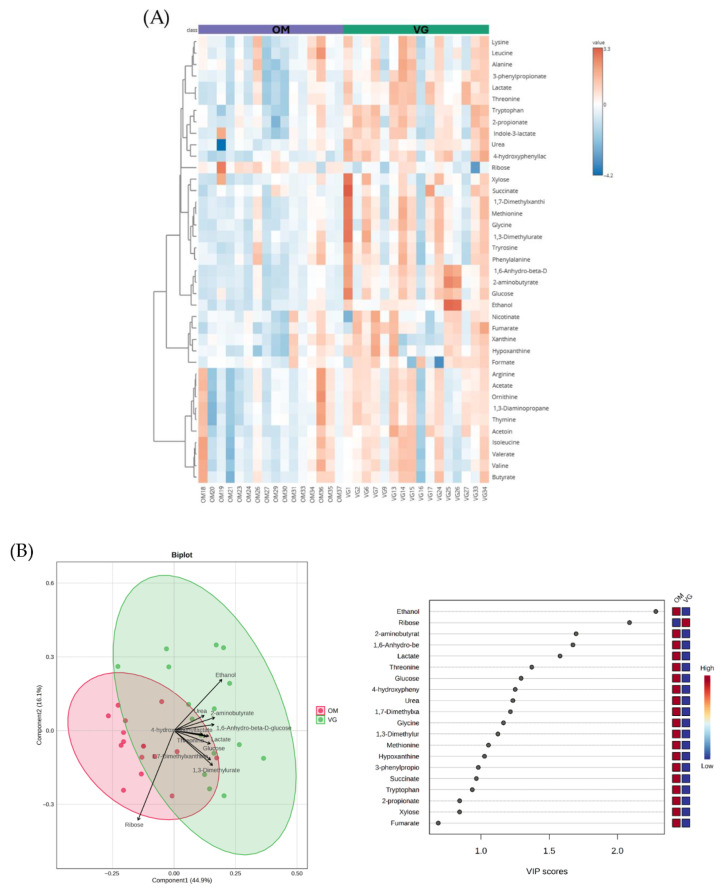
The untargeted metabolomics analysis of fecal samples between the vegetarian (VG) and omnivore (OM) groups. (**A**) Heatmap analysis of untargeted metabolomics analysis. (**B**) Partial Least Squares Discriminant Analysis (PLS-DA; biplot) and VIP score analysis of untargeted fecal metabolites.

The untargeted metabolomics analysis of fecal samples corroborated these functional differences. The VG group exhibited higher levels of 41 metabolites, including amino acids, fatty acids, organic acids, nucleobases/vitamins, and sugars (Figure 5A). Interestingly, in Figure 5B, ribose was the only metabolite elevated in the OM group.

### 3.3. Microbial Diversity and Functional Pathways of Different Fermented Foods

**Figure 6:**
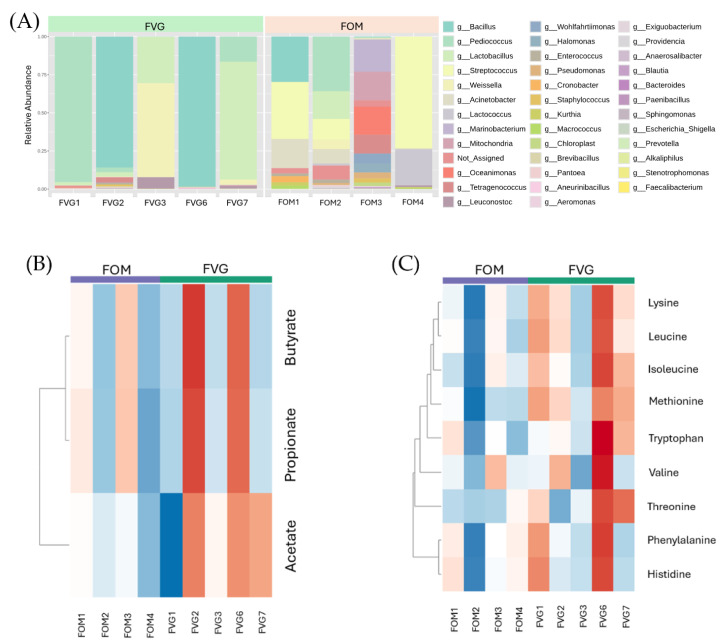
Microbial composition and metabolite profiles of fermented foods consumed by vegetarian (FVG) and omnivorous (FOM) groups. (**A**) Relative abundance of bacterial genera identified in fermented foods consumed by the vegetarian (FVG) and omnivorous (FOM) groups. (**B**) Heatmap analysis of short-chain fatty acid profile in fermented foods. (**C**) Heatmap analysis of essential amino acid profile in fermented foods. Color intensity represents relative concentration, where blue indicates low concentration and red indicates high concentration.

The dominant microbial communities identified in these foods are shown in Figure 6A and Table 1. Among the FVG samples, sweetened fermented rice (Khao-Mak; FVG-1), was dominated by *Pediococcus* (95.3%) and *Lactobacillus* (2.11%), while the salty fermented soybean condiment (plara-jae; FVG-2) and salty fermented soybean paste (kapi-jea: FVG-6) were rich in *Bacillus* (88.0% and 98.1%, respectively). The sour fermented mushroom (nham: FVG-3), mainly contained *Weissella* (61.8%) and *Lactobacillus* (30.0%), whereas sour fermented spinach (FVG-7) included *Lactobacillus* (77.2%), *Pediococcus* (16.5%), and *Weissella* (3.4%).

In contrast, FOM samples exhibited greater microbial diversity. Fermented soybean curd (FOM-1) was dominated by *Streptococcus* (73.0%) and *Bacillus* (12.9%), sour fermented spider flower (pak sian dong, FOM-2) contained *Pediococcus* (34.3%), *Lactobacillus* (18.9%), *Streptococcus* (14.7%), and *Weissella* (6.5%), salty fermented fish (plara, FOM-3) harbored marine and halophilic bacteria including *Marinobacterium* (20.4%), *Oceanimonas* (17.9%), *Tetragenococcus* (12.0%), *Halanaerobium* (8.2%), and *Halomonas* (5.8%), and kefir (FOM-4) was mainly *Streptococcus* (73.2%) and *Lactococcus* (23.6%).

To further elucidate the functional roles of fermented-food-associated microbes, targeted ^1^H-NMR-based metabolomic analysis was performed on the food samples to quantify key microbial-derived metabolites, particularly SCFAs and EAAs.

As shown in Figure 6B, the SCFA profiles differed significantly between the FVG and FOM groups. In the FVG group, fermented plant-based foods with *Bacillus*, such as FVG-2 and FVG-6, as well as fermented fiber-based foods rich in lactic acid bacteria (LAB) including FVG-1, FVG-3, and FVG-7, exhibited similar concentrations of SCFAs (acetate, propionate, and butyrate), which were significantly higher than all FOM samples. The observed difference was relatively higher but not statistically significant. Among the FOM samples, salty fermented protein animal-based foods showed higher concentrations of FOM-3 than others.

Regarding EAAs, the FVG samples also exhibited higher overall concentrations compared to FOM samples (Figure 6C). Specifically, all FVG fermentations except FVG-3 (mushroom-based food), showed elevated levels of leucine, isoleucine, valine, lysine, and threonine. Among these, FVG-6, a salty fermented soybean-based food dominated by *Bacillus*, showed the highest EAA concentrations.

### 3.4. Fermented Food Consumption Pattern Impact on Gut Bacterial Microbiota and Function

**Figure 7:**
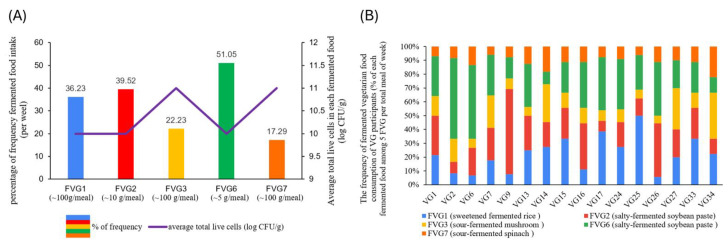
Frequency of fermented vegetarian food consumption among vegetarian (VG) participants. (**A**) Overall consumption frequency of the fermented vegetarian food, expressed as the percentage of total weekly meals. The purple line indicates the average total viable microbial counts (log CFU/g) detected in each fermented food. (**B**) Distribution of fermented vegetarian food consumption across individual VG participants, expressed as the relative contribution of each fermented food to total weekly fermented food intake.

Fermented food consumption frequency differed markedly between groups. OM participants reported infrequent intake (<10%/week), whereas VG participants consumed fermented foods daily, often in multiple forms (average > 60% of total meals per week) (Figure 7). Among all foods, FVG-6 was the most frequently consumed product (51.05% per week among the five FVGs), typically used as a salty condiment (~5 g/meal with an estimated 55 g intake per week), followed by FVG-2 as a dipping sauce (~10 g/meal with an estimated 80 g intake per week), FVG-1 as a sweet snack (~100 g/meal, with an estimated 760 g intake per week), and FVG-3 and FVG-7 as main dishes (~100 g/meal each with an estimated 500 and 400 g intake per week). Based on both consumption frequency, portion size, and dominant microbial composition, the genera most consistently ingested by VG participants could be *Pediococcus, Weissella*, *Lactobacillus* and *Bacillus,* respectively (Table 2 and Table S1).

Comparison of VG gut bacterial microbiota with FVG communities revealed 12 shared bacterial genera, although their relative abundances differed considerably (Table 3 and Table S2). Fermented food bacterial communities were compositionally distinct from VG gut microbiota; however, fermentation-associated genera such as *Lactobacillus*, *Pediococcus*, and *Weissella,* which are predominant in FVG samples, were similarly observed in the VG gut bacterial microbiota, at relatively low abundances. In contrast, these genera were present at a low abundance or not detected in the OM group.

## 4. Discussion

The gut bacterial microbiota diversity within individual groups (alpha diversity) demonstrated that long-term dietary patterns shape gut microbial diversity and composition in postmenopausal women. Participants were habitual consumers of fermented foods; fecal samples reflect the gut bacterial community under long-term dietary exposure rather than changes following a controlled intervention. Consequently, the findings should be interpreted as associations related to habitual dietary patterns rather than causal effects of fermented food intake. Gut bacterial microbiota from the OM group exhibited significantly higher phylum-level alpha diversity than VG, while genus-level alpha diversity remained stable, suggesting broader taxonomic distribution rather than changes in lower richness, and the stable genus-level indices indicate conserved community evenness across diets. In contrast, beta diversity demonstrates significantly distinct microbial community structures between the VG and OM groups, indicating that dietary patterns exert a notable influence on overall gut microbial community structure.

Bacterial taxonomic profiling of gut bacterial microbiota revealed distinct microbial compositions despite shared dominance of Bacteroidota and Firmicutes. Higher Bacteroidota abundance was observed in the VG group, whereas relatively greater Firmicutes abundance was found in the OM group. At the genus level, taxa enriched in the VG group were commonly associated with high-fiber, plant-based diets and fermentation of complex carbohydrates, producing beneficial short-chain fatty acids (SCFAs) such as acetate, propionate, and butyrate, while the OM group reflected adaptation to diets richer in animal-derived proteins and fats [30,31]. All these genera are normal gut bacteria, although *Escherichia–Shigella* includes strains that can be opportunistic under certain conditions.

LEfSe analysis was performed to identify genera that distinctly characterize each dietary group. The VG group was characterized by genera consistent with plant-based, high-fiber diets. In contrast, the OM group exhibited genera often linked to higher intake of animal-based foods and fats, some of which have been associated with gut inflammation or dysbiosis in previous studies [30,31,32]. These findings collectively indicate that long-term dietary patterns significantly shape the gut bacterial composition in postmenopausal women, influencing both the structure and specific microbial biomarkers of the gut bacterial microbiota.

KEGG pathway enrichment further revealed diet-related functional differences in the gut bacterial microbiota. Pathways enriched in the VG group were typically associated with plant-derived nutrients and complex carbohydrate fermentation, suggesting that the VG group gut bacterial microbiota may have adapted to efficiently utilize plant-based substrates and synthesize essential micronutrients. For instance, enrichment of riboflavin metabolism could indicate enhanced microbial production of B vitamins in response to lower dietary intake from animal sources, while zeatin biosynthesis reflects microbial capabilities related to plant hormone metabolism, which may influence host–microbe interactions. In contrast, the OM group exhibited enrichment of pathways related to protein and lipid metabolism, emphasizing amino acid catabolism and energy extraction from diverse dietary substrates. Notably, the increased capacity for branched-chain amino acid degradation in the OM group could reflect higher protein consumption, which has been linked to metabolites associated with metabolic health and, in some cases, insulin resistance.

The untargeted ^1^H-NMR-based metabolomic analysis of fecal samples corroborated these functional differences. Increased SCFAs and amino acids in the VG group could indicate enhanced microbial synthesis and fermentation activity, contributing to microbial metabolic activity through the production of metabolites such as SCFAs that support intestinal barrier function and modulate inflammation [30,32,33]. Interestingly, ribose was the only metabolite elevated in the OM group, which may reflect differences in carbohydrate availability or microbial preference in the omnivorous gut environment [34].

Overall, the functional KEGG pathway and metabolite data indicate that long-term dietary patterns strongly shape gut bacterial microbiota functionality and fecal metabolite profiles. Vegetarian diets promote pathways and metabolites associated with plant-based nutrient metabolism and SCFA production, whereas omnivorous diets favor amino acid degradation, energy extraction from proteins, and certain nucleotide-related metabolites. These findings underscore the dynamic relationship between diet, bacterial microbiota function, and metabolite output, which may have effects on host metabolic and immune health. Most of the bacterial genera identified in this study align with previous reports on diet-driven gut bacterial microbiota [7,33,35]. Fermented foods differ from unfermented plant foods because they contain microbially transformed substrates, fermentation-derived metabolites, and viable microorganisms generated during the fermentation process [33]. These components may influence nutrient availability and microbial metabolism after ingestion and may interact with the resident gut microbial community. The observed microbial shifts, including the enrichment of beneficial taxa such as *Prevotella* and *Weissella*, likely reflect the combined influence of a fiber-rich plant-based diet and the consumption of plant-based fermented foods. The enrichment of *Prevotella* observed in the VG group may largely reflect the high-fiber plant-based diet, which provides substrates for microbial fermentation. Nevertheless, plant-based fermented foods may contribute additional microbial metabolites and viable microorganisms that potentially modulate these fermentation processes and influence gut microbial activity [36,37]. A few findings may be distinctive; *Weissella* was significantly enriched in the VG group. In addition, this genus is less frequently reported as a dominant gut microbe in adults, suggesting that its enrichment may be related to the habitual daily intake of food containing live microbes. In this study, the VG participants consumed fermented foods daily at over 100 g/day, providing live microbes exceeding 10^8^ cells, which underscores the need to evaluate potential relationships between fermented food consumption and gut bacterial composition and function. These observations suggest that habitual dietary habits, particularly fermented food consumption, may shape unique aspects of the gut microbiota in postmenopausal women, which were further investigated in this study.

When microbial diversity in fermented foods was examined, the dominant microbial communities identified in FVG samples, particularly *Pediococcus* and *Lactobacillus*, were dominant in fiber-based fermented food, and *Weissella* was significantly prevalent in fermented mushroom, while spore-forming *Bacillus* was dominant in salty fermented plant protein. Notably, all genera found in all types of fermented foods in this study could possess fiber-degrading enzymes such as β-glucosidase, xylanase, and cellulase, enabling breakdown of plant polysaccharides into fermentable substrates, promoting SCFA (acetate, propionate, butyrate) and EAA production [33,38,39,40]. These metabolites serve as readily available nutrient molecules that promptly support gut and metabolic health.

In FOM, only one fermented food (salty fermented soybean-based food) is similar to FVG, although it was produced through slightly different manufacturing processes. *Streptococcus* was found predominant, while *Bacillus* was observed as minor bacteria in this food. Salty fermented protein animal-based food contained greater microbial diversity, including marine bacteria *Streptococcus*, *Marinobacterium*, and *Halomonas*, likely reflecting differences in raw materials and fermentation environments. Fermented dairy-based food contained mainly lactic acid bacteria.

The functional roles of fermented food-associated microbes were investigated by targeted ^1^H-NMR-based metabolomic analysis. In FOM samples, the substrates contain complete nutrition molecules that are richer to support the growth of a wide range of microorganisms. Consequently, in this fermentation ecosystem, the microbial communities were more diverse with no single predominant SCFA-producing species, potentially leading to less efficient and specific fermentation toward SCFA production as observed in FVG.

In contrast, FVG samples, rich in dietary fiber as the main carbon source and particularly fermentable plant fibers, provided a selective environment that supported the dominance of fiber-degrading bacteria, particularly LAB genera such as *Lactobacillus*, *Pediococcus*, and *Weissella*. These bacteria are capable of metabolizing complex carbohydrates and generating metabolites such as lactate, which can subsequently be utilized by other gut microbes through cross-feeding interactions to produce short-chain fatty acids (SCFAs), potentially contributing to the higher metabolite levels observed.

In this study, dietary fiber intake was estimated using the INMUCAL-Nutrients V4 database, which provides values for total dietary fiber only. Based on previous research [41,42], spinach and oyster mushrooms contain predominantly insoluble fractions generally characterized by lower fermentability; the VG group exhibited significantly higher fecal SCFA levels, suggesting that microbial fermentation in the gut may not depend solely on the soluble fiber fraction.

The high-fiber nature of these plant-based fermented foods likely promotes a *Prevotella*-dominant microbiota, consistent with findings in populations consuming high-fiber diets [43]. In addition, intestinal microbes possess diverse enzymatic systems capable of degrading complex plant polysaccharides, leading to the production of SCFAs [44]. Mushroom-derived β-glucans may serve as fermentable substrates for certain SCFA-producing bacteria and may contribute to the increased production of acetate, propionate, and butyrate observed in the VG cohort [41].

Furthermore, the fermentation process itself plays a crucial role in modifying plant cell wall structures and complex polysaccharides. Fermented foods differ from unfermented plant foods because they contain microbially transformed substrates and fermentation-derived metabolites. Lactic acid fermentation may act as a partial “pre-digestion” step that increases microbial accessibility to plant polysaccharides once they reach the colon, potentially enhancing their fermentability compared with unfermented plant foods [33]. Additionally, fermented soybean-based foods exhibited relatively higher SCFA concentrations compared to fiber-based and mushroom-based foods. This may be due to the presence of lipids in soybeans, which can be hydrolyzed by certain microbes into SCFAs [33,45], thereby enhancing the overall SCFA yield in these samples over carbohydrate-based and mushroom-based foods, as SCFA metabolites were synthesized by certain strains [46].

Regarding EAAs, the FVG samples also exhibited higher overall EAA concentrations compared to FOM samples. Soybean, as a plant-based protein substrate that provides a complete protein, contains all nine EAAs but a lower EAA content than animal-based proteins on a gram basis [47]. Interestingly, some reports demonstrated the function of a fermentable bacterial strain through a metabolic pathway converting carbohydrates in soybean to EAA. Thus, this report proposed an analysis of soybean fermentation and found that post-fermentation analysis revealed the presence of high concentrations of EAAs in fermented soybean samples through intermediates of central carbon metabolism, including glycolysis (e.g., phosphoenolpyruvate and pyruvate) and the TCA cycle [48]. Key biosynthetic pathways involved include the biosynthesis of amino acids (KEGG map 01230), the aspartate family pathway, the shikimate pathway (for aromatic amino acids), and the sulfur amino acid pathway for methionine production.

Overall, SCFA and EAA production in FOM samples was lower and less consistent than in FVG samples. These findings indicate that plant-based fermented foods harbor microbes with stronger functional potential for fiber fermentation and amino acid biosynthesis, whereas animal-based fermented foods exhibit broader microbial diversity with variable metabolic output. From this study, it could be confirmed that the fermentation process plays important roles in the transformation of plant substrates to complete EEAs profiles as well as significantly higher amounts of SCFAs. So, this finding implies that FVGs could be unintentional supplements that contain both essential nutritional potentiallytial beneficial microbes for this VG group.

Fermented food consumption frequency differed markedly between groups based on both consumption frequency, portion size, and dominant microbial composition. VG participants are repeatedly exposed to plant-associated microbes, potentially facilitating their transient transfer to the gut. Such habitual consumption may therefore represent a key ecological driver in determining microbial flux between food and host, unlike OM participants. Thus, only the gut bacterial microbiota of the VG and FVG were selected for further investigation.

Comparison of VG gut bacterial microbiota with FVG communities: *Weissella* was significantly more abundant in the group. Notably, it was not observed in the OM group, suggesting a dietary origin. In addition, previous reports support that *Weissella* was not reported as typical flora in human gut bacterial microbiota, although this bacterium was detected in human feces, the researchers confirmed that it derived from fermented vegetable consumption [47,49,50]. Therefore, habitual consumption of fiber-rich fermented foods may provide a continuous dietary source of beneficial microorganisms, including probiotic-associated bacteria. Although many of these microbes may behave as transient members of the gut microbiota, repeated intake may facilitate their ongoing introduction into the gut ecosystem, potentially supporting microbial balance and contributing to microbial metabolic activity, including the production of metabolites such as SCFAs and EAAs. In contrast, *Pediococcus* abundance did not differ significantly between the VG and OM groups, implying that its presence may not be diet-dependent, or that ingested strains have no probiotic potential for survival and persistence in the gastrointestinal tract. This finding underscores the distinction between transient carriage and true colonization, while some food-associated microbes may be temporarily detectable in fecal samples, only a subset may exert physiological effects or successfully integrate into the resident gut community [33,51,52].

The absence of *Bacillus* in VG gut samples, despite its prevalence in FVG-6, may reflect both low ingestion levels (due to consumption as condiment) and less survival of vegetative cells under gastrointestinal stress. Nevertheless, *Bacillus-*driven fermentation contributes valuable metabolites, including SCFAs and EAAs, which enrich the nutritional profile of these foods. Thus, even without gut colonization, fermented vegetarian foods may act as dietary vehicles delivering bioactive metabolites and transient microbes that influence host nutrient metabolism. These results provide new insight into the functional role of fermented foods in vegetarian diets; beyond acting as microbial carriers, fermented foods may serve as a dietary source of metabolites and microorganisms that interact with the gut microbial ecosystem that generate health-promoting metabolites, thereby supporting nutrient adequacy and potentially mitigating some of the nutritional limitations associated with long-term vegetarian dietary patterns. From this study, several potential microbes, particularly *Bacillus*, *Weissella*, and *Pediococcus*, demonstrated the ability to transform plant-based substrates into SCFA-rich fermented products, which may support gut health, modulate immune responses, and enhance metabolic functions. Remarkably, these microbes also contributed to the biosynthesis of EAAs in fiber-based foods [18,48,53], effectively enriching them with complete amino acid profiles that are typically limited in vegetarian diets. The selective microbial strains could be applied to develop functional plant-based fermented foods that nutritionally complement vegetarian diets, avoiding animal-based components. Moreover, they provide information for the development of novel probiotics such as *Weissella*, capable of colonizing the gut and enhancing metabolite production, including that of SCFA and EAA, to promote nutritional adequacy and long-term health in vegetarians.

This study is among the early pilot investigations examining the gut bacterial microbiota of postmenopausal women adhering to a long-term vegan diet with habitual consumption of fermented foods containing live microorganisms. Such foods may provide a continuous dietary source of microorganisms commonly associated with fermented foods. Although many of these microbes may behave as transient members of the gut microbiota, repeated intake may facilitate their continual introduction into the gut environment and they may transiently interact with the resident microbial community. This population and dietary context remain underrepresented in nutrition and bacterial microbiota research. By focusing on regular intake of home-prepared fermented foods, the study reflects realistic dietary behavior and exposure to viable microbes. The findings suggest that microbial metabolism associated with fermented food intake may contribute to the presence of health-related metabolites, including short-chain fatty acids and amino acids, some of which are commonly considered at risk of insufficiency in vegan diets. Increased dietary fiber intake associated with plant-based diets may also contribute to the observed metabolite profiles. Dietary fiber provides fermentable substrates for gut bacteria, stimulating microbial fermentation and the production of metabolites such as short-chain fatty acids. Thus, the metabolic patterns observed in the VG group may partly reflect higher fiber availability in the gut environment. *Weissella* is generally not considered a typical member of the core human gut microbiota [50] and is more commonly associated with fermented foods and plant-associated environments. Fermented foods may therefore act as a vehicle for introducing food-associated microorganisms into the human gastrointestinal tract [50,54]. Accordingly, the detection of *Weissella* in fecal samples may reflect dietary introduction through fermented food consumption rather than stable colonization.

However, studies specifically investigating the wash-out dynamics of *Weissella* in the human gut are currently limited. This genus is generally not considered a typical member of the core human gut microbiota and is more commonly associated with fermented foods and plant-associated environments [50]. Therefore, its detection in fecal samples may reflect dietary introduction through fermented food consumption rather than stable colonization. Several studies have shown that probiotic strains detected in fecal samples during supplementation often decline rapidly after cessation of intake, returning to baseline levels within a relatively short period. Tremblay (2023) [55] reported that most probiotic strains persisted in the fecal microbiome for only approximately 3–6 days after intake cessation, except Bifidobacterium longum R0175, which persisted longer (approximately 15 days). Similarly, Zmora et al. (2018) [56] reported that probiotic colonization in the human gut is highly individualized and often temporary. In their study, probiotic strains were detectable during supplementation but were largely absent from fecal samples following a wash-out period of approximately one month, regardless of whether individuals exhibited permissive colonization or resistance to probiotic engraftment. These observations provide baseline data to support further nutritional- and bacterial microbiota-focused investigations and may help inform the future development of targeted functional foods.

A limitation of this study is the limited number of participants who met the strict inclusion and exclusion criteria. Eligible participants were required to have a normal body mass index, be free from metabolic and cardiovascular diseases, and not use related medications. In addition, participants were further screened based on fermented food consumption frequency while limiting the number of eligible individuals within the same geographic area, which further limited the number of eligible individuals. Nevertheless, the final sample size was determined based on statistical power analysis despite these limitations. In addition, microbial profiling was performed using 16S rRNA gene sequencing, which characterizes bacterial taxa but does not capture other microbial kingdoms such as fungi, viruses, or archaea. Functional pathway analysis was inferred using PICRUSt, which provides predicted functional potential rather than direct measurement of metabolic activity. Finally, the cross-sectional design and single-timepoint sampling limit the ability to infer causal relationships, and the relatively small cohort may restrict the generalizability of the findings.

Future studies using shotgun metagenomics, metatranscriptomics, and multi-kingdom microbial profiling (including fungi and the virome) may provide a more comprehensive understanding of microbial community structure and function. In addition, studies with larger cohorts, longitudinal sampling, and controlled dietary interventions will be necessary to further validate the associations observed in this study and clarify potential relationships between fermented food consumption, gut bacterial microbiota modulation, and metabolic outcomes.

## 5. Conclusions

In summary, the microbial and metabolic signatures identified in the gut of healthy postmenopausal VG and OM participants suggest that long-term dietary patterns may influence gut microbial composition and metabolic activity through microbiota–metabolite interactions. Functional predictions and fecal metabolomics indicated that the VG gut bacterial microbiota was associated with carbohydrate fermentation and short-chain fatty acid production, whereas OM profiles reflected protein catabolism. In addition, *Weissella* was identified as a bacterial taxon significantly enriched in VG participants.

The frequency and portion size of fermented food consumption in the VG group may have contributed to the presence of microbial taxa commonly associated with fermented foods, including *Lactobacillus*, *Pediococcus*, and particularly *Weissella*. In addition, the fermented foods also contained high levels of SCFAs and a full spectrum of essential amino acids. These findings indicate that plant-based fermented foods harbor microbes with stronger functional potential for fiber fermentation and amino acid biosynthesis, whereas animal-based fermented foods exhibit broader microbial diversity with more variable metabolic outcomes. Overall, these findings could be key information in the promotion of development strategies for healthy postmenopausal women that require further investigation, particularly the definition of key health indicators such as blood biomarkers.

## Figures and Tables

**Table 1:** Nutrition information of each fermented food from VG and OM group.

ID	Fermented Food	Process	Nutritional Information					
			CHO (g)	FAT (g)	PRO (g)	PROA (g)	PROV (g)	ENER (kcal/100 g)
FVG1	fermented rice (sweet snack)	Ready to eat	39.70	4.55	3.80	0.00	3.80	206.95
FVG2	salty fermented soybean condiment (dipping sauce)	Ready to eat	34.64	7.92	15.58	0.00	15.58	272.16
FVG3	sour fermented mushroom (main dish)	Ready to eat	27.39	0.35	3.78	0.00	3.78	127.87
FVG6	salty fermented soybean paste (condiment)	Ready to eat	34.10	12.20	7.91	0.00	7.91	337.00
FVG7	sour fermented spinach (main dish)	Ready to eat	57.18	3.31	3.78	0.00	3.78	273.66
FOM1	fermented soybean curd (accompaniment)	Ready to eat	9.23	5.29	11.21	0.00	11.21	129.36
FOM2	sour fermented spider flower (main dish)	Ready to eat	8.43	3.20	4.00	0.00	4.00	78.52
FOM3	salty fermented fish (condiment)	Ready to eat	2.47	4.06	12.97	12.97	0.00	98.33
FOM4	kefir (drink)	Ready to eat	12.03	3.04	4.51	4.51	0.00	93.52

**Table 2:** The percentage of dominant bacterial genera in each fermented food.

Range	FVG1	FVG2	FVG3	FVG6	FVG7
**80–100%**	*Pediococcus*	*Bacillus*		*Bacillus*	
**60–80%**			*Weissella*		*Lactobacillus*
**40–60%**					
**20–40%**			*Lactobacillus*		
**0–20%**	*Bacillus*, *Lactobacillus*, *Weissella*, *Cronobacter*, *Pantoea*, *Exiguobacterium*, *Paenibacillus*, *Acinetobacter*, *Staphylococcus*	*Lactobacillus*, *Pediococcus*, *Weissella*, *Aneurinibacillus*, *Brevibacillus*, *Enterococcus*, *Tetragenococcus*, *Halanaerobium*, *Halomonas*, *Acinetobacter*, *Proteus*, *Alkaliphilus*, *Anaerosalibacter, Prevotella*, *Faecalibacterium*, *Staphylococcus*, *Wohlfahrtiimonas*	*Leuconostoc*, *Bacillus*, *Enterococcus*, *Exiguobacterium*, *Pediococcus*, *Acinetobacter*, *Kurthia*, *Prevotella*, *Pseudomonas*, *Staphylococcus*, *Lactococcus*	*Aneurinibacillus*, *Brevibacillus*, *Escherichia_Shigella*, *Anaerosalibacter*, *Faecalibacterium*	*Bacteroides*, *Enterobacteriaceae, Blautia*, *Pediococcus*, *Leuconostoc*, *Weissella*, *Prevotella*, *Faecalibacterium*, *Staphylococcus*, *Lactococcus*, *Streptococcus*

**Table 3:** Bacterial genera in VG gut bacterial microbiota and their fermented food.

Genera Bacterial	Average of VG Gut Bacterial Microbiota (%)	Average of FVG Bacterial Communities (%)	Average of OM Gut Bacterial Microbiota (%)	Average of FOM Bacterial Communities (%)
*f__Prevotellaceae_g__Prevotella*	29.646 ± 0.271	0.034 ± 0.001	9.011 ± 0.146	0.914 ± 0.017
*f__Bacteroidaceae_g__Bacteroides*	15.806 ± 0.169	0.011 ± 0.000	17.255 ± 0.179	2.261 ± 0.045
*f__Ruminococcaceae_g__Faecalibacterium*	11.943 ± 0.073	0.026 ± 0.000	11.375 ± 0.071	0.008 ± 0.000
*f__Lachnospiraceae_g__Blautia*	3.637 ± 0.029	0.026 ± 0.001	6.627 ± 0.032	2.010 ± 0.040
*f__Enterobacteriaceae_g__Escherichia_Shigella*	1.212 ± 0.022	0.020 ± 0.000	5.673 ± 0.095	0.275 ± 0.004
*f__Leuconostocaceae_g__Weissella*	0.612 ± 0.009	13.119 ± 0.273	0.033 ± 0.001	4.013 ± 0.079
*f__Streptococcaceae_g__Streptococcus*	0.556 ± 0.007	0.009 ± 0.007	0.933 ± 0.013	55.575 ± 0.375
*f__Lactobacillaceae_g__Pediococcus*	0.126 ± 0.004	23.005 ± 0.004	ND	ND
*f__Enterococcaceae_g__Enterococcus*	0.075 ± 0.001	0.242 ± 0.001	0.186 ± 0.004	0.745 ± 0.008
*f__Leuconostocaceae_g__Leuconostoc*	0.013 ± 0.000	1.814 ± 0.000	ND	ND
*f__Streptococcaceae_g__Lactococcus*	0.010 ± 0.000	0.124 ± 0.000	0.015 ± 0.000	7.291 ± 0.116
*f__Lactobacillaceae_g__Lactobacillus*	0.004 ± 0.000	22.588 ± 0.000	0.008 ± 0.000	1.193 ± 0.024

*f*: Family level and *g*: genus level. ND: not detected. Data are presented as mean ± standard deviation (SD).

## Data Availability

The original contributions presented in the study are included in the article/Supplementary Materials, further inquiries can be directed to the corresponding author.

## Supplementary Materials

The following supporting information can be downloaded at https://www.mdpi.com/article/10.3390/foods15071210/s1, Figure S1: LefSe analysis of each significant bacterial genera (A) VG was significantly higher than OM (B) OM was significantly higher than VG group at *p* < 0.05; Table S1: The dominant bacterial genera in each fermented food; Table S2: The dominant bacterial genera in each VG participant.

## Abbreviations

The following abbreviations are used in this manuscript:

ACE	Abundance-based coverage estimator
ANFs	Antinutritional factors
ASVs	Amplicon sequence variants
BMI	Body mass index
EAAs	Essential amino acids
FOM	Fermented food samples from omnivore group
FOM-1	Fermented soybean curd
FOM-2	Sour fermented spider flower (pak sian dong)
FOM-3	Salty fermented fish
FOM-4	Kefir
FVG	Fermented food samples from vegetarian group
FVG-1	Sweetened fermented rice (Khao-Mak)
FVG-2	Salty fermented soybean condiment (plara-jae)
FVG-3	Sour fermented mushroom (nham)
FVG-6	Salty fermented soybean paste (kapi-jea)
FVG-7	Sour fermented spinach
KEGG	Kyoto Encyclopedia of Genes and Genomes pathways
LDA	Linear Discriminant Analysis
LEfSe	Linear Discriminant Analysis Effect Size
NMR	Nuclear magnetic resonance
OM	Omnivore
OTUs	Operational Taxonomic Units
PCoA	Principal Coordinate Analysis
LAB	Lactic acid bacteria
PERMANOVA	Permutational Multivariate Analysis of Variance
rRNA	Ribosomal RNA
SCFAs	Short-chain fatty acids
STAMP	Statistical Analysis of Metagenomic Profiles
VG	Vegetarian
