# Supplementary material for: Diet-Associated Gut Bacterial Microbiota and Metabolome Signatures Linked to Fermented Food Intake in Healthy Postmenopausal Women
Source: Foods. 2026 Apr 2;15(7):1210. doi: 10.3390/foods15071210 (PMC13073002; doi:10.3390/foods15071210)
Supplement: Supplementary file 1 [file foods-15-01210-s001.zip › foods-4174405-supplementary.pdf]

Supplementary

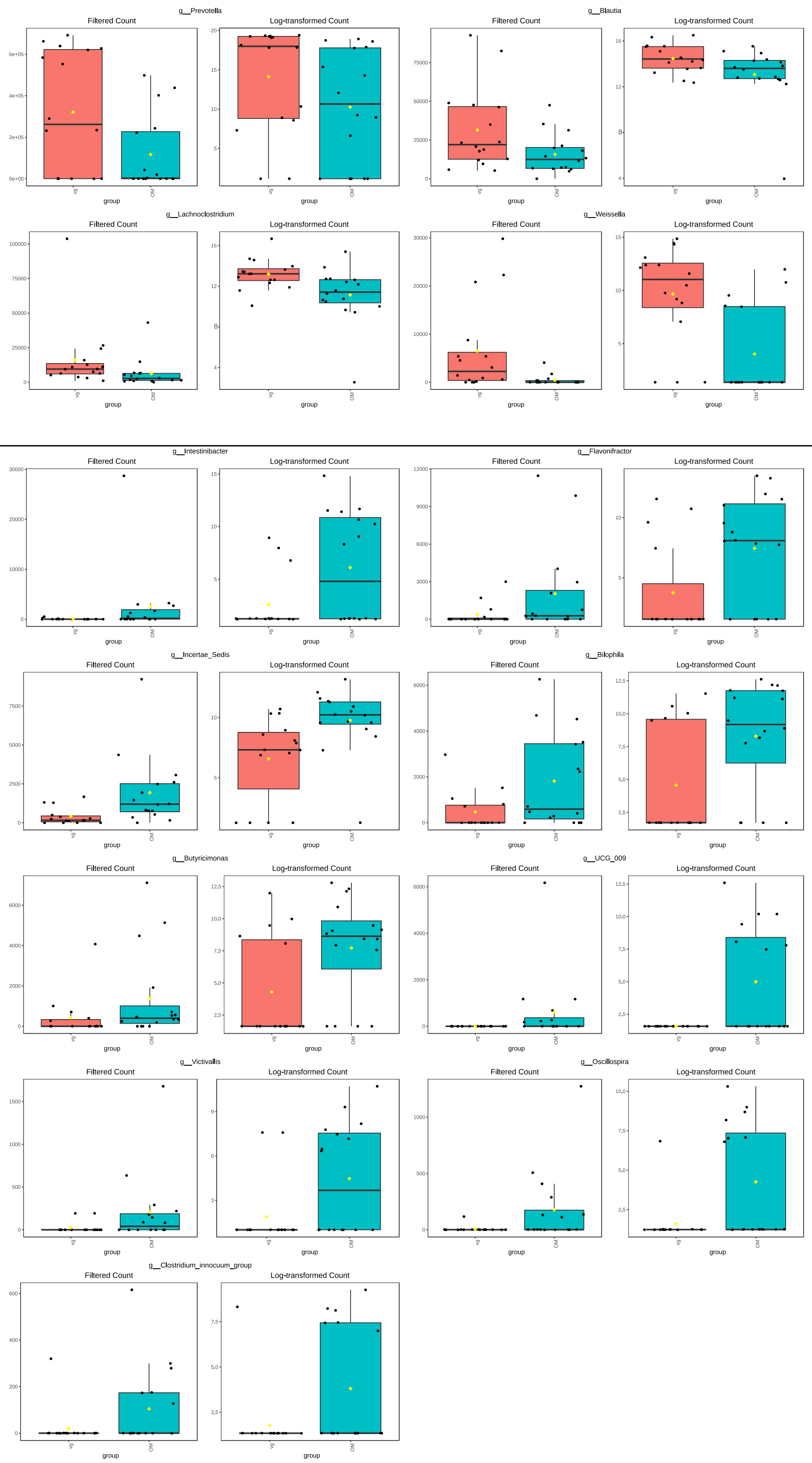

Figure S1 LefSe analysis of each significant bacterial genera A) VG was significantly higher than OM B) OM was significantly higher than VG group at $p < 0.05$

Table S1 The dominant bacterial genera in each fermented food

Genera bacterial	FVG1 (%)	FVG2 (%)	FVG3 (%)	FVG6 (%)	FVG7 (%)
NA	0.279	0.000	0.000	0.000	0.000
f__Aeromonadaceae_g__Aeromonas	0.000	0.000	0.000	0.000	0.000
f__Aneurinibacillaceae_g__Aneurinibacillus	0.000	0.268	0.000	0.751	0.000
f__Bacillaceae_g__Bacillus	0.155	85.088	0.019	98.248	0.000
f__Bacteroidaceae_g__Bacteroides	0.000	0.000	0.000	0.000	0.053
f__Brevibacillaceae_g__Brevibacillus	0.000	1.096	0.000	0.926	0.000
f__Comamonadaceae	0.000	0.101	0.000	0.000	0.000
f__Enterobacteriaceae	1.167	0.086	0.000	0.000	0.297
f__Enterobacteriaceae_g__Cronobacter	0.153	0.000	0.000	0.000	0.000
f__Enterobacteriaceae_g__Escherichia_Shigella	0.000	0.000	0.000	0.023	0.000
f__Enterococcaceae_g__Enterococcus	0.000	1.008	0.203	0.000	0.000
f__Enterococcaceae_g__Tetragenococcus	0.000	3.367	0.000	0.000	0.000
f__Erwiniaceae_g__Pantoea	0.587	0.000	0.000	0.000	0.000
f__Exiguobacteraceae_g__Exiguobacterium	0.021	0.000	0.028	0.000	0.000
f__Halanaerobiaceae_g__Halanaerobium	0.000	0.205	0.000	0.000	0.000
f__Halomonadaceae_g__Halomonas	0.000	0.127	0.000	0.000	0.000
f__Lachnospiraceae_g__Blautia	0.000	0.000	0.000	0.000	0.129
f__Lactobacillaceae_g__Lactobacillus	2.109	3.578	30.034	0.000	77.218
f__Lactobacillaceae_g__Pediococcus	95.299	3.027	0.200	0.000	16.497
f__Leuconostocaceae_g__Leuconostoc	0.000	0.000	7.420	0.000	1.653
f__Leuconostocaceae_g__Weissella	0.077	0.309	61.839	0.000	3.368
f__Mitochondria_g__Mitochondria	0.012	0.000	0.000	0.000	0.000
f__Moraxellaceae_g__Acinetobacter	0.075	0.117	0.007	0.000	0.000
f__Morganellaceae_g__Proteus	0.000	0.130	0.000	0.000	0.000
f__Morganellaceae_g__Providencia	0.000	0.000	0.000	0.000	0.000
f__Nitrincolaceae_g__Marinobacterium	0.000	0.013	0.000	0.000	0.000
f__Paenibacillaceae_g__Paenibacillus	0.016	0.000	0.000	0.000	0.000
f__Peptostreptococcales_Tissierellales_ g__Alkaliphilus	0.000	0.094	0.000	0.000	0.000
f__Peptostreptococcales_Tissierellales_ g__Anaerosalibacter	0.000	0.512	0.000	0.023	0.000
f__Planococcaceae_g__Kurthia	0.000	0.000	0.048	0.000	0.000
f__Prevotellaceae_g__Prevotella	0.000	0.029	0.017	0.000	0.125
f__Pseudomonadaceae_g__Pseudomonas	0.000	0.000	0.020	0.000	0.000

f__Ruminococcaceae_g__Faecalibacterium	0.000	0.023	0.000	0.029	0.077
f__Staphylococcaceae_g__Macrococcus	0.000	0.000	0.000	0.000	0.000
f__Staphylococcaceae_g__Staphylococcus	0.049	0.769	0.057	0.000	0.029
f__Streptococcaceae_g__Lactococcus	0.000	0.000	0.109	0.000	0.513
f__Streptococcaceae_g__Streptococcus	0.000	0.000	0.000	0.000	0.043
f__Wohlfahrtiimonadaceae_g__Wohlfahrtiimonas	0.000	0.052	0.000	0.000	0.000
f__Xanthomonadaceae_g__Stenotrophomonas	0.000	0.000	0.000	0.000	0.000

f; family level and g; genus level

Table S2 The dominant bacterial genera in each VG participant

Genera bacterial	VG1	VG2	VG6	VG7	VG9	VG13	VG14	VG15	VG16	VG17	VG24	VG25	VG26	VG27	VG33	VG34
f__Prevotellaceae_g__Prevotella	54.665	24.525	0.047	51.459	52.239	65.200	0.000	24.643	64.567	57.161	0.000	58.140	0.130	21.498	0.020	0.035
f__Bacteroidaceae_g__Bacteroides	1.778	19.961	30.779	0.346	0.362	0.476	29.333	14.483	2.849	2.741	22.083	2.762	42.565	4.806	54.438	23.127
f__Ruminococcaceae_g__Faecalibacterium	8.420	22.457	25.232	10.238	19.236	8.306	1.303	5.882	6.390	17.469	16.607	12.338	3.944	19.777	3.821	9.677
f__Lachnospiraceae_g__Blautia	1.492	0.899	4.574	1.284	1.044	2.507	11.012	2.914	0.593	3.344	8.798	2.476	3.507	4.932	2.972	5.837
f__Enterobacteriaceae	4.087	0.981	0.326	3.322	16.281	1.618	2.384	0.464	8.079	0.823	0.139	1.370	2.321	1.916	0.036	5.973
f__Lachnospiraceae_g__Lachnospiraceae_NK4A136_group	0.971	2.179	15.513	3.416	1.655	1.321	0.272	2.112	2.246	1.593	2.519	3.514	0.014	3.193	1.552	2.579
f__Ruminococcaceae_g__Subdoligranulum	1.111	7.268	2.170	1.187	0.188	0.764	4.619	0.405	0.827	0.979	4.397	4.235	0.000	1.171	2.228	2.432
f__Lachnospiraceae	1.026	4.690	0.254	1.461	0.859	1.029	2.237	2.337	0.365	1.941	1.707	1.469	0.243	6.396	3.130	2.298
f__Lachnospiraceae_g__Lachnoclostridium	0.898	1.006	0.742	0.480	0.088	0.634	1.214	1.148	0.297	0.407	0.701	1.180	10.386	2.497	3.503	1.567
f__Bifidobacteriaceae_g__Bifidobacterium	0.165	0.738	0.595	0.000	0.000	1.629	4.864	3.495	0.045	1.157	9.915	0.104	0.535	1.096	0.904	0.721
f__Lachnospiraceae_g__Roseburia	0.843	0.442	1.994	3.149	0.388	0.775	0.258	4.175	0.473	1.330	0.588	0.229	4.863	1.638	0.107	3.966
f__Rikenellaceae_g__Alistipes	0.099	1.463	0.382	0.112	0.000	0.015	12.091	0.667	0.642	0.175	3.662	0.091	0.000	0.324	1.720	3.012
f__Sutterellaceae_g__Sutterella	0.000	2.770	0.973	0.108	1.164	1.025	0.000	5.323	0.800	0.000	3.150	2.468	4.283	0.980	0.000	1.091

f__Enterobacteriaceae_g__Escherichia_Shigella	8.189	0.043	0.086	0.595	0.163	0.147	0.472	0.185	1.501	3.593	0.019	0.151	4.428	0.114	1.997	1.276
f__Acidaminococcaceae_g__Phascolarctobacterium	2.925	0.670	1.269	0.245	0.844	1.165	0.000	6.291	2.291	0.979	1.377	0.000	0.000	0.656	0.902	2.863
f__Lachnospiraceae_g__Eubacterium_eligens_group	1.690	0.473	0.158	0.556	0.237	0.891	0.424	3.098	0.958	0.229	0.000	0.000	5.830	0.309	1.984	2.592
f__Selenomonadaceae_g__Megamonas	2.156	0.000	0.000	0.000	0.000	0.000	0.146	0.000	0.000	0.000	0.000	0.000	12.090	0.112	3.351	0.000
f__Oscillospiraceae_g__UCG_002	0.268	0.079	0.331	3.369	0.185	0.362	3.920	2.612	0.851	0.062	0.215	0.264	0.014	0.932	1.252	0.483
f__Christensenellaceae_g__Christensenellaceae_R_7_group	0.150	0.951	2.164	0.545	0.043	0.272	0.000	2.621	0.105	0.105	0.243	0.350	0.000	6.095	0.142	1.354
f__Lachnospiraceae_g__Lachnospiraceae_ND3007_group	0.330	0.077	0.808	0.339	0.271	0.255	4.926	0.811	0.348	1.044	2.576	0.886	0.974	0.405	0.246	0.352
f__Lachnospiraceae_g__Anaerostipes	0.422	0.886	0.507	0.087	0.034	0.345	0.378	1.162	0.137	0.407	0.860	0.558	0.466	0.461	0.221	6.938
f__Lachnospiraceae_g__Ruminococcus_torques_group	0.198	0.335	0.387	0.310	0.200	0.498	0.443	0.270	0.887	0.034	0.496	0.099	0.000	1.461	4.370	1.215
f__Leuconostocaceae_g__Weissella	2.086	0.011	0.000	0.501	0.037	1.962	0.443	0.572	0.139	0.054	0.885	0.285	0.000	2.736	0.000	0.081
f__Lachnospiraceae_g__Ruminococcus_gauvreauii_group	0.092	0.433	0.271	1.288	0.117	0.419	0.189	1.072	0.139	0.076	0.326	0.341	0.000	0.928	2.065	2.022
f__Lachnospiraceae_g__Lachnospira	0.033	0.283	0.061	1.190	0.043	0.250	0.123	0.000	0.463	0.000	2.208	1.314	1.272	1.248	0.000	1.106

f__Tannerellaceae_g__Parabacteroides	0.106	0.349	0.301	0.072	0.074	0.121	3.105	1.130	0.188	0.085	0.470	0.207	0.751	0.237	1.789	0.311
f__Streptococcaceae_g__Streptococcus	0.414	0.029	1.487	0.072	2.231	1.596	0.162	0.329	0.040	0.023	0.475	0.212	0.066	0.724	0.000	1.043
f__Ruminococcaceae_g__Ruminococcus	0.026	2.147	0.982	0.000	0.000	0.470	1.324	0.275	0.000	0.042	1.100	0.000	0.000	0.019	0.000	1.668
f__Lachnospiraceae_g__Coprococcus	0.125	0.351	0.362	1.331	0.000	0.173	0.281	0.243	0.081	0.000	2.427	0.143	0.000	1.609	0.269	0.443
f__Oscillospiraceae_g__UCG_003	0.000	0.226	0.480	0.848	0.000	0.090	1.586	0.513	0.247	0.272	0.895	0.000	0.000	0.828	0.000	0.000
f__Lachnospiraceae_g__Eubacterium_hallii_group	0.418	0.290	0.400	0.180	0.097	0.514	0.725	0.176	0.175	0.263	0.298	0.475	0.487	0.558	0.460	0.443
f__Lachnospiraceae_g__Eubacterium_ruminantium_group	0.000	0.000	0.000	0.765	0.000	0.259	2.064	0.000	0.000	0.000	0.623	0.203	0.000	0.837	0.000	0.972
f__Sutterellaceae_g__Parasutterella	0.169	0.036	0.041	0.000	0.011	0.000	4.681	0.045	0.030	0.045	0.309	0.022	0.000	0.025	0.030	0.000
f__Peptostreptococcaceae_g__Romboutsia	1.224	0.000	1.028	0.685	0.026	0.656	0.000	0.081	0.034	0.192	0.966	0.000	0.000	0.218	0.000	0.000
f__Ruminococcaceae_g__CAG_352	0.308	0.231	0.000	0.310	0.000	0.167	0.000	1.189	0.023	0.042	0.524	0.022	0.000	1.034	0.064	0.964
f__Pasteurellaceae_g__Haemophilus	0.279	0.016	1.648	0.516	0.379	0.612	0.000	0.104	0.073	0.011	0.217	0.177	0.030	0.386	0.000	0.111
f__Clostridiaceae_g__Clostridium_sensu_stricto_1	0.462	0.000	0.000	0.462	0.000	0.388	0.000	0.000	0.505	0.000	0.000	0.000	0.000	2.209	0.000	0.000
f__Lachnospiraceae_g__CAG_56	0.000	0.000	0.973	0.000	0.000	0.000	0.000	1.108	0.034	0.659	0.000	0.000	0.000	0.000	0.440	0.678

f__Lachnospiraceae_g__Lachnospiraceae_UCG_001	0.444	0.077	0.070	0.202	0.043	0.094	0.254	0.000	0.021	0.000	0.000	0.229	0.000	0.085	0.000	2.369
f__Erysipelatoclostridiaceae_g__Erysipelotrichaceae_UCG_003	0.092	0.156	0.211	0.029	0.023	0.009	0.185	0.518	0.294	0.252	0.000	0.138	0.000	0.081	0.307	1.539
f__Oscillospiraceae_g__NK4A214_group	0.092	0.066	0.000	0.743	0.000	0.149	0.000	0.140	0.000	0.000	1.733	0.056	0.000	0.212	0.013	0.000
f__Veillonellaceae_g__Veillonella	0.315	0.000	0.075	1.483	0.048	0.140	0.006	0.014	0.000	0.000	0.090	0.035	0.239	0.021	0.000	0.316
f__Prevotellaceae_g__Paraprevotella	0.000	0.000	0.661	0.000	0.080	0.000	0.000	0.910	0.633	0.252	0.000	0.000	0.000	0.000	0.000	0.000
f__Lachnospiraceae_g__Marvinbryantia	0.000	0.041	0.249	0.317	0.000	0.446	0.089	0.041	0.000	0.642	0.000	0.069	0.000	0.340	0.000	0.000
f__Lactobacillaceae_g__Pediococcus	0.000	0.000	0.000	1.659	0.040	0.000	0.181	0.000	0.053	0.000	0.000	0.000	0.000	0.000	0.089	0.000
f__Lachnospiraceae_g__Anaerosporobacter	0.000	0.000	0.000	0.079	0.000	0.031	0.000	0.095	0.000	0.000	1.561	0.173	0.000	0.052	0.000	0.000
f__Lachnospiraceae_g__Lachnospiraceae_UCG_004	0.000	0.208	0.190	0.155	0.000	0.000	0.000	0.000	0.000	0.235	0.293	0.000	0.371	0.062	0.132	0.121
f__Oscillospiraceae_g__UCG_005	0.000	0.000	0.156	0.815	0.037	0.097	0.156	0.176	0.066	0.011	0.000	0.117	0.000	0.058	0.000	0.000
f__Eggerthellaceae_g__uncultured	0.033	0.000	0.000	0.491	0.000	0.123	0.000	0.189	0.019	0.000	0.413	0.238	0.000	0.131	0.008	0.000
f__Lachnospiraceae_g__Lachnospiraceae_UCG_010	0.070	0.000	0.000	0.209	0.000	0.068	0.000	0.221	0.000	0.034	0.000	0.086	0.000	0.152	0.239	0.435
f__Clostridia_UCG_014_g__Clostridia_UCG_014	0.000	0.023	0.000	0.061	0.000	0.061	0.112	0.351	0.000	0.000	0.000	0.156	0.000	0.295	0.000	0.445

f__Ruminococcaceae_g__Eubacterium_siraeum_group	0.033	0.041	0.023	0.141	0.000	0.000	0.000	0.658	0.000	0.054	0.357	0.035	0.000	0.164	0.000	0.000
f__Oscillospiraceae_g__uncultured	0.000	0.104	0.023	0.000	0.000	0.000	0.303	0.356	0.000	0.000	0.170	0.000	0.000	0.000	0.158	0.311
f__Lachnospiraceae_g__Lachnospiraceae_FCS020_group	0.088	0.029	0.039	0.112	0.037	0.083	0.073	0.063	0.000	0.263	0.142	0.000	0.000	0.367	0.053	0.000
f__Eggerthellaceae_g__Adlercreutzia	0.000	0.018	0.020	0.000	0.000	0.000	0.445	0.140	0.006	0.000	0.000	0.000	0.000	0.000	0.064	0.651
f__Enterococcaceae_g__Enterococcus	0.000	0.014	0.047	0.314	0.000	0.000	0.328	0.000	0.224	0.243	0.000	0.000	0.027	0.000	0.000	0.000
f__Lachnospiraceae_g__Eubacterium_xylanophilum_group	0.000	0.163	0.000	0.047	0.000	0.000	0.008	0.225	0.000	0.000	0.149	0.000	0.000	0.100	0.000	0.478
f__Lachnospiraceae_g__uncultured	0.037	0.000	0.039	0.198	0.029	0.050	0.000	0.000	0.064	0.000	0.012	0.186	0.000	0.193	0.168	0.086
f__Lachnospiraceae_g__Lachnospiraceae_UCG_008	0.015	0.023	0.090	0.285	0.043	0.114	0.000	0.158	0.053	0.074	0.090	0.000	0.000	0.066	0.000	0.023
f__Desulfovibrionaceae_g__Bilophila	0.000	0.000	0.000	0.076	0.000	0.000	0.000	0.180	0.104	0.000	0.307	0.000	0.000	0.056	0.000	0.147
f__Lachnospiraceae_g__Eubacterium_ventriosum_group	0.000	0.072	0.000	0.025	0.020	0.011	0.042	0.239	0.000	0.000	0.071	0.000	0.025	0.021	0.130	0.165
f__Eubacterium_coprostanoligenes_group_g__Eubacterium_coprostanoligenes_group	0.000	0.000	0.000	0.000	0.009	0.000	0.187	0.032	0.105	0.017	0.165	0.000	0.000	0.054	0.051	0.000
f__Ruminococcaceae_g__uncultured	0.040	0.000	0.000	0.000	0.000	0.000	0.012	0.000	0.000	0.008	0.045	0.017	0.000	0.025	0.000	0.451

f__Lachnospiraceae_g__Agathobacter	0.000	0.000	0.000	0.000	0.000	0.075	0.191	0.099	0.000	0.000	0.047	0.078	0.000	0.077	0.000	0.000
f__Ruminococcaceae_g__Incertae_Sedis	0.015	0.011	0.034	0.036	0.000	0.011	0.000	0.000	0.092	0.000	0.000	0.026	0.000	0.000	0.109	0.000
f__Oxalobacteraceae	0.029	0.245	0.022	0.000	0.000	0.000	0.000	0.000	0.017	0.000	0.000	0.000	0.000	0.019	0.000	0.000
f__Atopobiaceae_g__uncultured	0.055	0.000	0.000	0.029	0.000	0.000	0.062	0.059	0.000	0.000	0.045	0.026	0.000	0.035	0.000	0.000
f__Lachnospiraceae_g__GCA_900066575	0.040	0.011	0.000	0.054	0.046	0.042	0.000	0.000	0.000	0.000	0.000	0.000	0.000	0.000	0.000	0.035
f__Peptococcaceae_g__Peptococcus	0.011	0.000	0.000	0.032	0.000	0.013	0.000	0.077	0.047	0.000	0.000	0.039	0.000	0.000	0.000	0.000
f__Leuconostocaceae_g__Leuconostoc	0.048	0.000	0.000	0.000	0.000	0.097	0.031	0.000	0.000	0.000	0.000	0.026	0.000	0.000	0.000	0.000
f__Lachnospiraceae_g__Moryella	0.037	0.000	0.016	0.051	0.000	0.018	0.019	0.000	0.000	0.000	0.000	0.000	0.000	0.019	0.018	0.000
f__Streptococcaceae_g__Lactococcus	0.029	0.000	0.000	0.000	0.000	0.037	0.000	0.000	0.000	0.000	0.000	0.000	0.000	0.006	0.000	0.091
f__Anaerovoracaceae_g__Family_XIII_UCG_001	0.037	0.000	0.000	0.025	0.000	0.022	0.000	0.000	0.008	0.000	0.000	0.000	0.000	0.012	0.000	0.000
f__Erysipelotrichaceae_g__Holdemania	0.000	0.000	0.000	0.011	0.000	0.000	0.023	0.000	0.000	0.000	0.017	0.000	0.000	0.012	0.010	0.025
f__Lactobacillaceae_g__Lactobacillus	0.000	0.000	0.000	0.058	0.011	0.000	0.000	0.000	0.000	0.000	0.000	0.000	0.000	0.000	0.000	0.000

f; family level and g; genus level
